# Higher vaccination rates predict reduction in SARS-CoV-2 transmission across the United States

**DOI:** 10.1007/s15010-022-01802-1

**Published:** 2022-03-22

**Authors:** Jacky Au

**Affiliations:** School of Education, University of California, Irvine, 3200 Education Building, Irvine, CA 92697 USA

**Keywords:** COVID-19, SARS-CoV-2, Pandemic, Vaccines

## Abstract

**Purpose:**

The severe acute respiratory syndrome coronavirus 2 (SARS-CoV-2) began proliferating widely throughout the world in late 2019/early 2020, creating a global pandemic and health crisis. Although vaccines became available to the public approximately 1 year after the onset of the pandemic, there still remains much hesitancy surrounding vaccination. One key concern comes from reports of breakthrough infections among the vaccinated that show comparable levels of peak viral load as the unvaccinated, calling into question the ability of vaccines to prevent transmission. Therefore young, healthy individuals who are at low risk of serious complications themselves have little incentive to receive a vaccine that they are not convinced will protect others around them. To address this important concern, this study aimed to evaluate the extent to which vaccination rates are associated with reduced SARS-CoV-2 transmission among the unvaccinated population.

**Methods:**

An observational study was conducted in the United States of America throughout the months of June through September, 2021. Vaccination rate and incidence of coronavirus disease 2019 (COVID-19) were obtained for each state, along with a number of important control variables. Panel data regression was used to predict incidence among the unvaccinated based on each state’s vaccination rate.

**Results:**

States with a higher proportion of fully vaccinated individuals reported fewer new cases among the remaining unvaccinated population.

**Conclusion:**

These data add to accumulating evidence that COVID-19 vaccinations can indeed slow the spread of SARS-CoV-2, and are an important tool in society’s arsenal to put this pandemic behind us.

**Supplementary Information:**

The online version contains supplementary material available at 10.1007/s15010-022-01802-1.

## Introduction

The severe acute respiratory syndrome coronavirus 2 (SARS-CoV-2) causes a respiratory disease known as coronavirus disease 2019 (COVID-19). Symptoms include fever, difficulty breathing, loss of smell or taste, and a host of other ailments ranging from minor to severe. This is an urgent global health crisis, resulting in over 251 million infections and over 5 million deaths worldwide at the time of writing, with the United States alone contributing roughly 1/6 of these numbers (46.7 million infections and 758,000 deaths). Fortunately, the recent development and distribution of vaccines has done much to curtail this issue, with initial clinical trials showing remarkable efficacy in preventing hospitalizations and deaths [1, 2]. A few months after mass distribution of vaccines began in the United States, many states began to relax lockdown regulations such as removing mask requirements to allow some semblance of pre-pandemic life to resume. However, with the recent surge of new mutations such as the Delta variant and persistent vaccine hesitancy among a large portion of the population [3], this global health crisis is far from over. One prominent case in the highly vaccinated state of Massachusetts received national attention in July 2021 when an outbreak occurred in Barnstable county, despite the majority of infected individuals being fully vaccinated [4]. Moreover, the Centers for Disease Control (CDC) report on this case went on to find comparable levels of viral load in the nose and throat of vaccinated and unvaccinated individuals based on PCR cycle threshold values, which indicates the possibility that even vaccinated individuals carry a significant risk of transmitting the virus [4].

Based in large part on the results of this report, the CDC went on to adjust their guidance to re-recommend universal indoor masking. This series of news events, along with the CDC’s response, has caused understandable concern among many and has raised questions regarding the true extent to which vaccines prevent individuals from contracting and spreading the virus. Claims have even arisen among those opposed to or hesitant about vaccines that vaccination only serves to increase the spread of SARS-CoV-2 since vaccinated individuals are running around with comparable viral load to the unvaccinated but fewer symptoms to indicate the presence of infection. Despite the abundance of evidence that has since come out suggesting that the vaccinated actually have lower overall infection rates, both symptomatic and asymptomatic [5–7] as well as faster recovery times that shorten the window of infectiousness [8, 9], such beliefs about vaccine ineffectiveness still persist and stymie mass vaccination efforts [10]. Although evidence that vaccines protect the individual from severe illness and hospitalizations [1, 2, 7, 11] is generally accepted and relatively non-controversial, there has been less focus on the effectiveness of vaccines in preventing transmission to others [12, but see 13]. However, this is a critical and timely issue to address, especially for the young and healthy unvaccinated individuals who are at the lowest risk of serious health complications from SARS-CoV-2 but have the highest propensity to transmit to others [14].

The purpose of this report, therefore, is to conduct a large-scale, nation-wide analysis on the association between a state’s vaccination rate and the development of new COVID-19 cases (incidence) among the remaining unvaccinated population. To answer this question, I used empirical real-world data on COVID-19 incidence and vaccination rates provided by the CDC for each of the 50 states in the United States of America, as well as Washington D.C. For ease of readability, references to states or state-level analyses in the remainder of the.

manuscript will implicitly also include Washington D.C. Data were analyzed starting from June, approximately 1 month after vaccines became widely available to the general public in the United States, through the present (September 2021 at the time of writing). Furthermore, several important confounds were considered for inclusion in the model as controls (see Variable Selection in Methods), such as population density or willingness to comply with other pandemic policies that have been shown to reduce transmission such as staying at home [15, 16] or wearing masks in public [17, 18]. Testing frequency was also considered, since states that conduct more random testing of asymptomatic individuals would be more likely to report higher overall case numbers. However, perhaps the most important variable to control was previous incidence during the same months in the preceding year. Since the same state-specific idiosyncrasies that contributed to case load last year, such as political attitudes [19], tourist hotspots, regional climate [20], demographics [21], and many others likely also contribute to case load this year, controlling for previous incidence allows for the model to account for the aggregated variation from these state-specific idiosyncrasies without the risk of overfitting the model by including each one individually.

The main hypothesis of these analyses is that states with higher vaccination rates will also report fewer new COVID-19 cases, which would lend support to the idea that getting vaccinated can protect others as well as oneself. However, there are two other possible outcomes as well. There could be no relationship, which may indicate that the effect of vaccination on community transmission is too small or non-existent to be detected through the noise of the myriad other nation-wide factors that contribute to viral transmission. Or there could even be a positive relationship such that more vaccinated states report more new cases rather than less. This would lend support to the idea often promulgated by vaccine skeptics that vaccination does nothing to slow down spread or infection, but does alter the behaviors of the vaccinated by masking symptoms and making them more likely to resume life as normal.

## Methods

### Data aggregation

#### Primary variables

Data on vaccination rates^1^ and COVID incidence,^2^ the primary independent and dependent variables of interest, respectively, were obtained using publicly available datasets from the CDC. Vaccination rate was defined as the percentage of a state’s population that was fully vaccinated at the beginning of a particular month. The criteria to be fully vaccinated followed the guidelines of the CDC during the observation period, which included individuals with one shot of the Johnson and Johnson JNJ-78436735 vaccine or two shots of the Pfizer BNT162b2 or Moderna mRNA-1273 vaccines. Incidence was defined as the proportion of new cases that arose in a particular state in a particular month per 100,000 unvaccinated individuals. Incidence for New York and New York City were reported separately in the CDC dataset, but were aggregated together in the current analyses to represent the incidence for the entire state of New York. A small, but unknown, number of the cases recorded in the CDC dataset consisted of breakthrough infections among the vaccinated. Since the CDC only tracks severe breakthrough infections that lead to hospitalizations or deaths, and do not report these at the state level, it is impossible to disaggregate the number of breakthrough infections from the total number of new cases each month within this dataset. I therefore estimated breakthrough rates based on partial data provided elsewhere.^3^ Unfortunately, these data were only limited to the first half of 2021, and were largely unavailable for the months of interest (June through September). Moreover, data from half the states were not reported. Thus, the number of breakthrough infections in the current analyses is only a crude estimate, extrapolating from the rate of breakthrough infections in the beginning of the year, and applying mean substitution to the states that did not report data. Although these assumptions are tenuous at best, I argue that this makes very little difference to the overall analyses, as the overwhelming majority of new cases reported to the CDC occurred among the unvaccinated. On average, only 1.7% of new reported cases occurred among the vaccinated while 98.3% occurred among the unvaccinated, based on the 25 states that reported data in the first half of 2021. Thus, I argue that any range of plausible breakthrough infection rates represents just a drop in the bucket when subtracted from the much larger pool of unvaccinated cases. To substantiate this argument, I provide sensitivity analyses where I run Monte Carlo simulations using randomly generated breakthrough infection rates for each state ranging from 0.4 to 12%, varying in increments of 0.01%. These boundaries were chosen based on conservatively doubling the minimum and maximum reported breakthrough infection rates, to account for recent increases in transmissibility due to the surge of the Delta variant. These values also match with breakthrough infection rates reported in the literature, which range from 0.02 to 13.3% [22–28], but mostly skew towards the lower end especially when taken from a random sample rather than high-exposure groups such as health care workers. Thus, these are conservative estimates that are more likely to overestimate than underestimate the effect of breakthrough infections on the current analyses. 10,000 simulations were run.

Finally, after calculating incidence in the preceding manner, a *z* transformation was applied separately for each month. This transformation was critical to standardize values between months and make valid comparisons because of the surge in cases over time due to the increasing spread of the Delta variant and other factors [29]. Without this transformation, a spurious correlation would occur in which incidence would appear to rise even as vaccination rates increase over the summer.

#### Control variables

Previous incidence from 2020 was calculated exactly as above, with the exception that cases were taken as a proportion of the entire population of a given state rather than the unvaccinated population since vaccines were not available then. The remaining control variables were extracted from various sources as follows. Population density, as well as the total population of each state, were extracted from World Population Review^4^ using 2021 estimates, since the official US Census data for 2021 are not available at the time of writing. Testing frequency was measured as the number of PCR diagnostic laboratory tests performed in each state, excluding a minority of results from non-laboratory or point-of-care settings, expressed as a percentage of that state’s population.^5^ Mobility was determined via GPS data from mobile devices that tracked the number of individuals in each state who stayed home on a particular day, summed over the course of each month and expressed as a percentage of the total number of mobile phones tracked in that state.^6^ Finally, data on mask compliance were provided by the Delphi Research Group at Carnegie Mellon University, who in partnership with Facebook, administered massive daily surveys starting from September 2020 asking about whether an individual wore a mask most or all of the time in public over the last 5–7 days.^7^ On average, each day included roughly 3840 responses per state. The daily percentage estimates provided by the Delphi group were weighted by the demographic breakdown of a particular state to provide a representative sample from that particular state. I then weighted these daily estimates by the sample size of respondents on that day and averaged over the entire year from September 2020 to September 2021 to produce one time-invariant measure per state that reflected the general propensity to wear masks in public over the past year. This was done instead of calculating dynamic monthly estimates of mask use specifically for the months of June through September for two reasons. First, previous research has shown mask use to predict lower COVID-19 incidence in 2020 [17, 18], but mask use has dropped off considerably since the widespread availability of vaccines in 2021, especially among the vaccinated. Thus, this variable might be measuring different things in 2021 compared to 2020, and averaging over the entire year smooths out the signal. Second, doing so provided stronger correlations with the other variables, especially vaccination rate. A stronger correlation produces a more meaningful, and often more conservative, analysis because the regression partials out the correlated variable and estimates the unique variance of vaccination rate over and above mask usage.

### Variable selection

Although all control variables were selected on strong theoretical grounds, care was taken to avoid overfitting the model with too many unnecessary variables. Thus, an inclusion criterion was set such that a variable must be significantly correlated with either the dependent variable (incidence) or the primary independent variable (vaccination rate) to be included in the final model [30, 31]. A test of variance inflation factors (VIF) was also carried out to rule out multicollinearity. All variables were averaged across months to test their correlations during this model selection process.

### Statistical analysis

All statistical analyses were conducted using Stata version 13.0 (StataCorp, 2013). I first performed a series of simple ordinary least-squares regressions for each month from June, 2021 through September, 2021. The dependent variable was the untransformed COVID-19 incidence and the independent variable was vaccination rate. This analysis was conducted to establish the existence of a simple relationship between vaccination rate and incidence, uninfluenced by any other extraneous variables, as per previous recommendations [30].

After establishing this simple relationship across all months of interest, I re-analyzed the data with a single model, while controlling for potential confounds. To do so, I used a random effects panel regression using month-aggregated data as the within-unit estimator of time and state as the cross-sectional between-unit estimator, according to the following model:

Incidence_*it*_ = β_0_ + β_1_VaxRate_*it*_ + β_2-4_Month_*it*_ + β_5-7_VaxRate_it_*Month_*it*_ + β_8_Mask_*it*_ + β_9_Testing_*it*_ + β_10_Incidence 2020_*it*_ + u_*i*_ + ε_*it*_.

Where the dependent variable, Incidence_*it*_, refers to the z-transformed incidence for state *i* during month *t*. β_0_ represents the overall regression intercept, β_1_ represents the coefficient for vaccination rate, β_2-4_ represent a 3 × 1 vector of coefficients for each month dummy variable, omitting the first one, June, β_5-7_ similarly represent a 3 × 1 vector of coefficients for the interaction of vaccination rate and each month dummy, β_8_ represents the coefficient for mask usage, β_9_ represents the coefficient for testing frequency, and β_10_ represents the coefficient for 2020 incidence. u_*i*_ + ε_*it*_ represents the composite error term where u_*i*_ represents a random intercept for each state and ε_*it*_ is the idiosyncratic error of each state for each month. Hausman’s test was used to test the existence of a correlation between u_*i*_. and the other independent variables to determine the appropriateness of the random effects model.

## Results

### Descriptive statistics

Incidence and vaccination rates are reported in Table 1 for each state, along with overall summary statistics. Raw data for all other variables can be found in Supplementary Table S1

**Table 1:** Incidence and Vaccination Rates per State

State	June		July		August		September		Average	
	Vax %	Incidence	Vax %	Incidence	Vax %	Incidence	Vax %	Incidence	Vax %	Incidence
Alaska	39.40	196.4	43.00	962.6	45.50	2937.1	47.30	6349.4	43.80	2611.4
Alabama	29.20	155.9	32.70	1206.4	34.40	4108.5	38.40	2325.4	33.68	1949.0
Arkansas	31.20	369.7	34.30	1792.6	36.50	3324.4	41.80	2357.7	35.95	1961.1
Arizona	36.10	262.9	43.10	711.6	45.30	1937.3	48.00	1965.0	43.13	1219.2
California	43.30	142.7	50.00	924.3	53.00	2142.4	56.00	1478.4	50.58	1172.0
Colorado	45.40	340.1	52.00	587.0	54.50	1490.2	57.00	1970.3	52.23	1096.9
Connecticut	53.70	112.0	60.80	386.7	63.40	1394.6	66.20	1435.5	61.03	832.2
Washington D.C	46.40	98.5	52.30	332.3	54.90	1442.7	57.40	1734.7	52.75	902.1
Delaware	43.40	153.3	50.10	333.2	52.80	1788.6	55.40	2938.7	50.43	1303.4
Florida	39.20	388.0	45.90	2420.7	49.00	5624.2	53.20	2943.5	46.83	2844.1
Georgia	32.10	143.5	36.70	698.4	38.70	3261.9	41.90	2735.9	37.35	1709.9
Hawaii	47.80	181.6	51.80	616.5	53.60	3119.5	55.40	2418.1	52.15	1583.9
Iowa	43.90	134.8	48.00	380.3	49.60	1621.2	51.80	3041.6	48.33	1294.5
Idaho	32.80	230.8	36.10	480.0	37.50	1752.0	39.30	3144.3	36.43	1401.8
Illinois	40.20	124.9	46.30	433.1	48.60	1543.7	51.30	1679.1	46.60	945.2
Indiana	35.50	220.3	40.00	443.3	44.30	2252.5	46.40	2826.2	41.55	1435.6
Kansas	38.50	207.9	42.00	894.3	45.30	2314.5	48.40	2549.7	43.55	1491.6
Kentucky	38.50	199.5	43.60	825.4	45.70	4167.0	48.70	4672.0	44.13	2466.0
Louisiana	31.30	296.4	35.00	2088.9	37.00	4866.5	41.60	1849.1	36.23	2275.3
Massachusetts	53.80	90.8	61.80	394.2	64.00	1567.0	66.00	2193.9	61.40	1061.5
Maryland	48.20	75.4	56.10	238.7	58.90	1165.4	61.60	1498.0	56.20	744.4
Maine	54.40	197.4	61.50	266.7	63.50	1163.1	65.90	3085.2	61.33	1178.1
Michigan	42.20	106.6	47.20	244.0	48.90	897.3	50.60	1833.6	47.23	770.4
Minnesota	46.20	125.4	52.00	270.7	53.90	1384.6	56.00	2393.2	52.03	1043.5
Missouri	34.40	493.3	39.20	1611.5	41.50	2153	45.20	1775.4	40.08	1508.3
Mississippi	27.10	190.4	29.80	1158.0	34.50	4696.3	38.40	2665.7	32.45	2177.6
Montana	38.30	278.5	42.90	421.7	44.40	1728.7	46.00	3933.1	42.90	1590.5
North Carolina	36.20	175.8	41.90	749.1	43.80	2842.8	46.50	2780.0	42.10	1636.9
North Dakota	36.60	152.2	38.90	196.4	40.20	1344.2	41.70	3252.2	39.35	1236.3
Nebraska	42.40	103.6	47.80	410.8	49.60	1637.1	52.10	2345.0	47.98	1124.2
New Hampshire	49.20	106.9	56.10	205.9	58.30	1189.3	59.70	2235.9	55.83	934.5
New Jersey	48.90	155.4	55.30	401.5	58.50	1425.7	61.60	1833.4	56.08	954.0
New Mexico	47.90	215.7	54.60	540.9	57.30	2400.7	60.10	2400.9	54.98	1389.5
Nevada	37.00	469.2	42.10	1254.0	44.50	1854.4	48.00	1822.7	42.90	1350.1
New York	47.00	121.2	54.20	407.6	57.20	1528.2	60.30	1902.4	54.68	989.8
Ohio	40.30	127.6	44.80	269.2	46.60	1439.7	48.50	3145.7	45.05	1245.6
Oklahoma	33.80	185.4	38.50	1058.5	40.30	2920.2	44.00	2695.5	39.15	1714.9
Oregon	45.50	300.5	53.80	584.1	56.00	2903.0	58.10	2934.7	53.35	1680.6
Pennsylvania	43.70	128.5	49.70	206.4	52.50	1120.2	55.20	2220.3	50.28	918.8
Rhode Island	51.80	131.8	59.00	477.7	61.60	2016.6	64.90	2564.4	59.33	1297.6
South Carolina	33.80	147.0	38.80	695.3	40.70	3665.0	43.50	3991.2	39.20	2124.6
South Dakota	42.40	65.7	45.50	168.8	47.00	1424.0	49.30	2759.0	46.05	1104.4
Tennessee	31.80	118.1	35.50	732.6	39.20	4243.7	42.00	3782.1	37.13	2219.1
Texas	35.60	229.0	41.30	758.8	43.90	2643.5	47.60	2793.1	42.10	1606.1
Utah	32.50	388.5	37.50	845.6	44.80	1653.0	47.80	2468.5	40.65	1338.9
Virginia	45.30	104.8	51.90	391.2	54.70	1859.2	57.40	2695.3	52.33	1262.6
Vermont	56.40	60.5	65.70	221.4	66.80	1498.9	67.90	2358.9	64.20	1034.9
Washington	46.70	351.8	54.70	653.5	57.70	2611.7	60.20	2987.0	54.83	1651.0
Wisconsin	44.60	97.1	49.60	342.3	51.80	1604.5	54.10	2789.2	50.03	1208.3
West Virginia	34.00	196.1	37.30	283.4	39.10	2050.0	39.70	4681.3	37.53	1802.7
Wyoming	31.80	492.1	34.50	761.6	36.70	2644.9	38.70	4268.0	35.43	2041.6
Average (SD)	40.94 (7.26)	198.9 (110.4)	46.34 (8.60)	661.6 (485.2)	48.78 (8.52)	2281.65 (1089.58)	51.45 (8.28)	2696.07 (925.66)	46.88 (8.12)	1459.53 (492.32)

Vaccination rate (Vax %) and incidence are provided for each state for each month, as well as overall average for the entire observation period. Vax % is defined as the percentage of each state’s population that is fully vaccinated by the beginning of each month. Incidence is defined as the number of new unvaccinated cases per 100,000. Standard deviations (SD) are provided at the bottom in parentheses

### Correlations between variables

Table 2 shows a pairwise correlation matrix between the dependent and all independent variables considered in the model. Population density and mobility were dropped from the final model due to a lack of significant correlations with both incidence as well as vaccination rates (*p’s* > 0.081). All other variables were retained.

**Table 2:** Correlation Matrix

Variables	(1)	(2)	(3)	(4)	(5)	(6)	(7)
(1) Incidence	1.000						
(2) Mask usage	− 0.265 (0.060)	1.000					
(3) Testing frequency	− 0.116 (0.418)	0.472 (0.000)	1.000				
(4) Mobility	− 0.010 (0.490)	0.503 (0.000)	0.529 (0.000)	1.000			
(5) Population density	− 0.220 (0.121)	0.311 (0.026)	0.523 (0.000)	0.505 (0.000)	1.000		
(6) Incidence2020	0.479 (0.000)	− 0.376 (0.006)	− 0.437 (0.001)	− 0.334 (0.017)	− 0.148	1.000 (0.301)	
(7) Vaccination rate	− 0.611 (0.000)	0.603 (0.000)	0.452 (0.001)	0.248 (0.080)	0.183 (0.198)	− 0.753 (0.000)	1.000

Incidence is the dependent variable and vaccination rate is the primary independent variable of interest. *P* values are included in parentheses

Due to the significant correlations between the remaining variables, VIFs were tested to ensure multicollinearity was not present in the model. All variables had a VIF under 10–Vaccination rate: 3.19, 2020 Incidence: 2.48, Mask Rate: 1.78, Testing Frequency: 1.43, Mean VIF: 2.22.

### Simple linear regression

**Fig. 1:**
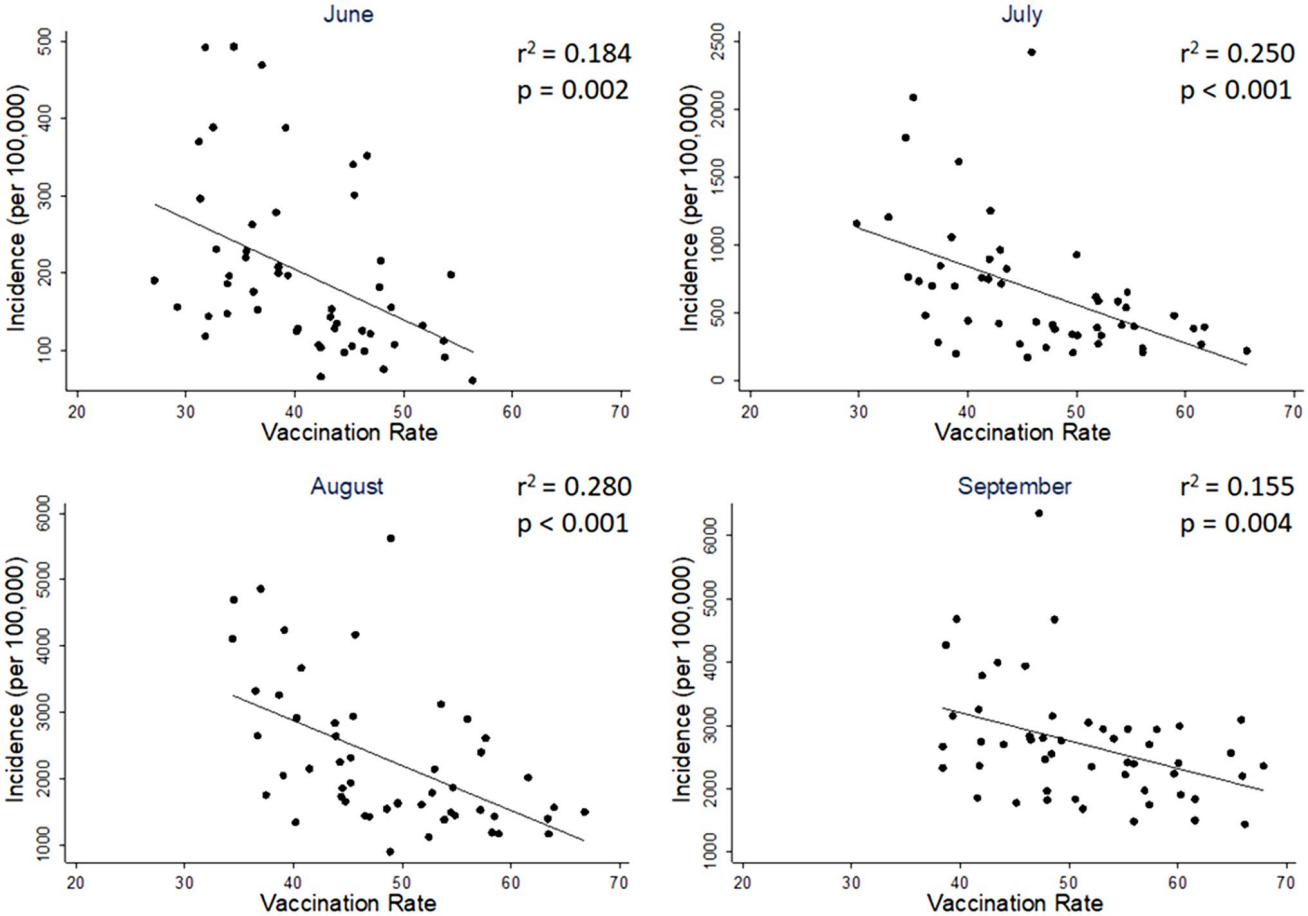
Vaccination rate predicts lower incidence. Significant negative associations between vaccination rate and COVID-19 incidence were found during the months of June through September, with vaccination rate explaining between 15.5 and 28% of the variance in COVID-19 incidence. For ease of interpretation, the y-axis is unstandardized in these figures, and left in its natural units (number of new cases per 100,000 unvaccinated people)

Simple ordinary least-squares linear regressions were run separately for each month, regressing incidence on vaccination rate (Fig. 1). To facilitate interpretation, the dependent variable was left untransformed in its natural units (cases per 100,000 unvaccinated people) for this analysis. All months showed significant negative associations: June (*b* = − 6.530, *p* = 0.002, *r*^2^ = 0.184), July (*b* = − 28.210, *p* < 0.001, *r*^2^ = 0.250), August (*b* = − 67.685, *p* < 0.001, *r*^2^ = 0.280), and September (*b* = − 44.010, *p* = 0.004, *r*^2^ = 0.155).

### Panel data regression

Panel data regression was used to confirm the effects of the simple linear regression (Table 3). Hausman’s test detected no significant endogeneity (*χ*^2^ (9) = 10.13, *p* = 0.340), and thus the random effects model was chosen over fixed effects. In concordance with the simple linear regression models, the random effects panel regression model showed a significant partial effect of vaccination rate (*b* = − 0.055, *z* = − 2.79, *p* = 0.005) referenced to the month of June, with no significant interactions for the months of July (*b* = 0.004, *z* = 0.18, *p* = 0.856), August (*b* = − 0.0001, *z* = 0.00, *p* = 0.996), or September (*b* = 0.003, *z* = 0.14, *p* = 0.890), suggesting comparable effects of vaccination on COVID-19 incidence across all months. Previous incidence rates from 2020 were also predictive of current incidence rates (*b* = 0.230, *z* = 2.73, *p* = 0.006), as was testing frequency (*b* = 0.028, *z* = 2.16, *p* = 0.031). The between R-squared value for the model was 0.399, suggesting almost 40% of the variation in COVID incidence between states can be explained by the variables in this model. Given the lack of interactions with month, I re-ran the above regression without the interaction terms to arrive at a main effect of vaccination rate across all months: (*b* = − 0.050, *z* = − 3.51, *p* < 0.001).

**Table 3:** Panel regression results

Independent variables	*b*	*SE b*	*p*
Vaccination rate	− 0.055	0.020	0.005*
July	0.132	0.870	0.879
August	0.273	0.905	0.763
September	0.200	0.941	0.832
Vaccination rate X July	0.004	0.020	0.856
Vaccination rate X August	0.000	0.020	0.996
Vaccination rate X September	0.003	0.020	0.890
Incidence2020	0.230	0.084	0.006*
Mask usage	0.011	0.014	0.423
Testing frequency	0.028	1.292	0.031*

The dependent variable is COVID-19 incidence, *z* transformed by month. Each independent variable is expressed as a percentage, so their regression coefficients (*b*) can be interpreted as standard deviation changes in response to a 1% change in the independent variable. The exceptions are Incidence 2020, which is also *z* transformed into standard deviation units, and the month dummies which are coded as 1’s and 0’s. June was omitted as the reference. *SE b* is the standard error of the regression coefficient.

*represents significant *p* value < 0.05

### Sensitivity analyses

Re-running the above panel regression using a range of different estimates for breakthrough infection rates did not change the results. Across 10,000 Monte Carlo simulations, the partial effect of vaccination rate for June ranged between − 0.049 and − 0.062 standard deviations, with all *p* values showing statistical significance ranging from *p* = 0.005 to *p* = 0.033. None of the interactions with any other month were significant (all *p*’s < 0.71) with coefficients ranging from − 0.001 to 0.007, suggesting comparable effects of vaccination rate across all months. The same held true looking at the main effect of vaccination rate by removing interaction terms. Over 10,000 simulations, the main effect of vaccination rate ranged between − 0.044 and − 0.056, with all p values again in the significance range, from *p* < 0.001 to *p* = 0.002.

## Discussion

The present analyses provide compelling evidence for the real-world effectiveness of COVID-19 vaccines in reducing community transmission of SARS CoV-2 in the United States. Despite rising cases overall throughout the summer months due to the Delta surge and other factors, higher vaccination rates in a state at the beginning of each month still predicted fewer cases during that month relative to other states. Critically, COVID-19 incidence was calculated as a proportion of the unvaccinated population, not a proportion of the total population. Thus, these results go beyond the already plentiful evidence that the various COVID-19 vaccines are generally effective at protecting vaccinated individuals from symptomatic infection [1, 2, 7, 11], but also provide evidence supporting the effectiveness of vaccines in protecting the surrounding unvaccinated community as well. Moreover, results held up against a variety of different specifications for breakthrough infection rates, which were subtracted from the total number of new cases before calculating incidence among the unvaccinated. Although these breakthrough rates were not reported in the CDC dataset used in the current analyses and had to be roughly estimated, they occur so infrequently and are reported even less often, that their presence makes very little difference in the overall analyses. This was substantiated by Monte Carlo simulations that randomly generated breakthrough infection rates for each state using a range of realistic values and found that not one of the 10,000 simulations changed the interpretation of the results or had any substantial effect on the regression estimates (± 0.006 standard deviations for the main effect of vaccination rate).

Throughout the observation period during the summer of 2021 (June through September), each percentage increase in a state’s vaccinated population was associated with a reduction in new COVID-19 cases by approximately 0.053 standard deviations. To illustrate the real world implications of this, Table 1 shows that one standard deviation throughout these months averaged to about 492.32 infections per 100,000 unvaccinated people. Thus, in a hypothetical population of 100,000 unvaccinated people, each 1000 (or 1%) of them who became fully vaccinated at the beginning of June would be associated with an average of 26.09 fewer cases per month among their unvaccinated peers. By the end of September, these same 1000 vaccinations would be associated with 104.37 fewer infections overall. More impressively, these results span a window of time during which the Delta variant was the predominant strain in the United States, suggesting that vaccines may be effective at slowing spread even against this highly infectious strain.

However, it is noteworthy that these results stand in contrast to another recent report using a similar approach that found no relationship between vaccination rate and COVID-19 incidence, both internationally and within the United States [32]. However, that study has a couple limitations that render interpretation difficult. One is that their observation period is only 7 days, so it is unclear how representative their data truly are of the entire year since vaccines have become available. This is especially problematic for their United States analysis where incidence was calculated at the county level. A number of counties in the United States have such small populations, in the hundreds or thousands, that a mere one-week observation period could result in nonrepresentative outliers. For example, many such counties could have realistically seen zero cases in that particular week, whereas others may have randomly experienced a severe outbreak that week, leading to an unusually high percentage of their population being infected. In contrast, the analyses in the current report which do find an effect of vaccination, aggregate data over 4 months and at the state-level, thereby smoothing out noise over time and a larger population size. Another limitation of this previous report is that their analyses were purely correlational and did not control for alternative predictors that may covary with either vaccination rate or COVID-19 incidence. To give an example from the current dataset, Table 2 shows that vaccination rate and testing frequency are both correlated (*r* = 0.452, *p* = 0.001). This is not surprising in that states that are proactive about vaccinating their citizens are also proactive about testing their citizens in an effort to control the spread. Obviously, conducting more tests will yield more positive cases, so without controlling for the covariation of testing frequency with vaccination rate, a spurious correlation could arise where regions with higher vaccination rates appear to have more new cases. Such controls become even more important at the international level, where so many different factors exist between countries such as vaccine type, cultural attitudes, and even climate [20] that could moderate the effect of vaccination on incidence in one direction or the other.

Accordingly, one of the strengths of the current results is that they are robust against a variety of important controls, including mask usage, testing frequency, and previous incidence. Therefore, alternative explanations relating to these variables can be ruled out. Most importantly, including previous incidence in the model serves as a powerful control because it effectively accounts for the aggregated variation due to state- and time-specific idiosyncrasies that may influence the endemic spread of SARS-CoV-2 within a particular state. For example, a state that reported a high case load last summer due to large gatherings at popular tourist beaches may also report a high case load this year due to the same beaches. Or another state typically known for its warm and humid summers may create inhospitable conditions for the virus to spread both last year and this [20]. In support of these idiosyncratic influences, Table 2 shows a fairly strong correlation between previous and current incidence (*r* = 0.48, *p* < 0.001). Thus, controlling for previous incidence accounts for the aggregated influences of these state- and time-specific factors. This is a particularly important consideration given that a random-effects model was used, which favors more precise coefficient estimates compared to a fixed effects model, but at the expense of possibly introducing some level of omitted variable bias due to correlations between the unit-level (i.e., state) error terms and the other independent variables [33]. Controlling for state-level idiosyncrasies partially mitigates this issue by reducing variation associated with state-level error, achieving some of the same goals as a fixed effects model in terms of offering some level of correction against omitted variable bias while maintaining the improved precision of a random effects model.

An additional and important consideration is that summer incidence during 2020 was related not only to summer incidence during 2021, but also had a strong negative correlation with the primary independent variable, vaccination rate (*r* = − 0.75, *p* < 0.001). Therefore, increased vaccination rate predicted lower incidence both this year and last. Ostensibly, this is problematic since vaccines clearly cannot have a retroactive effect; therefore, this independent variable must reflect more than just vaccination rate, but perhaps also the willingness to employ a constellation of other pandemic policies as well that may have influenced transmission over the last year. For example, Table 2 shows that states with higher vaccination rates this summer also wore masks more often throughout the last year (*r* = 0.603, *p* < 0.001), indicating that a part of the variation in vaccination rate (*r*^2^ = 0.364) overlaps with the variation in mask usage. Although there are likely other unmeasured variables at play, this is one important factor that could mediate the retrospective relationship between vaccination rate and 2020 incidence, especially given that mask usage has already been shown to predict lower COVID-19 incidence in 2020 [17, 18]. There are two important takeaways from all this. First is that vaccines are not the only tool in society’s arsenal to stem the tide of the pandemic. Although the present results say little about which specific policies and practices, other than vaccination, are helpful, the correlations do suggest that states that tend to favor vaccination also tend to favor other recommendations and guidelines, and ultimately tend to fare better. Second, it is important to bear in mind that these correlations do not take away from the main message of the manuscript, which is that vaccinations, specifically, predict lower COVID-19 incidence. Rather, the results of the panel regression show that the effect of vaccination rates is robust enough to still uniquely predict current incidence, even above and beyond its relationships with the aforementioned confounds.

Furthermore, these findings are in line with recent studies that show fewer symptomatic and asymptomatic infections among the vaccinated, as assessed by routine laboratory PCR testing [5–7, 34]. Thus, states with a more vaccinated populace have fewer vectors of transmission, so it is no surprise that they also seem to offer more protection from infection even to its unvaccinated residents. In addition, evidence also shows that aside from being less likely to host the virus in the first place, vaccinated individuals with breakthrough infections also carry less overall viral load throughout the duration of infection, despite having similar peak levels as the unvaccinated at the beginning of infection [8, 9, 35]. Moreover, even for the same levels of viral load, less infectious virus was found in respiratory samples among the vaccinated, indicative of less viral shedding [36, 37]. Therefore, while appreciating that breakthrough infections, as well as transmission, can and do occur among the vaccinated [8, 27, 28], several mechanisms seem to be at play that limit the spread of virus compared to the unvaccinated. Aside from the current study, this reduction in transmission has also been borne out empirically in other recent large-scale studies in Israel that showed reduced transmission from vaccinated individuals to their households compared to the households of unvaccinated individuals [12, 13].

### Limitations

The empirical results reported herein must be understood in the context of several limitations. First, due to the rapidly evolving nature of this virus [38], it cannot be certain that the protective effect of vaccination on the unvaccinated will hold true with future mutations. For example, although the observation window of this study is based primarily on the Alpha and Delta virus strains, another variant, Omicron, has since arisen with an even higher infectivity and vaccine resistance than its predecessors [38]. Although preliminary evidence suggests vaccination still reduces viral load in Omicron breakthrough infections to a comparable amount as Delta breakthrough infections [39], it is possible other mechanisms of infectivity exist and it is still unclear the extent to which vaccine-mediated reductions in transmission can be expected during the age of Omicron. Another limitation in the current study is the low granularity with respect to type of vaccination. Three main vaccines have been used in the United States, manufactured by Pfizer, Moderna, and Johnson and Johnson. However, due to the roughly homogenous uptake of all three vaccines between states, it is beyond the scope of the current study to determine their relative efficacy in reducing transmission.

## Conclusions

In totality, the evidence that COVID-19 vaccinations reduce the spread of SARS-CoV-2, at least among the Alpha and Delta variants, is quite strong and consistent. Although the public messaging from the government and other authorities so far has focused primarily on the effectiveness of COVID-19 vaccines in protecting the individual, enough evidence has accrued now that this message should shift to the effectiveness of vaccines in protecting the community. This message would be especially pertinent to young, healthy adults who are the least likely to suffer major complications if infected, and therefore have the least personal incentive to get vaccinated. Although the vaccination of older adults has done much to reduce hospital burden and save lives among the elderly, it is now the young and healthy that need to be prioritized for vaccination. In support of this, an enterprising study by Monod et al. [14] modeled infection dynamics across the nation and correlated this to cell phone mobility data. They found that the contact patterns of younger adults, aged 20–49 were most predictive of COVID-19 transmission and deaths than any other age group. Although vaccine mandates may be one viable solution to increase vaccination rates among younger adults across the country, this has been met by severe pushback from many, especially by those who consider vaccination to be solely a personal health choice. Considering that the strongest and most publicized evidence of vaccine effectiveness so far has been the reduction of hospitalizations and deaths among the individual [1, 2, 5, 7, 11, 40], and that the public health narrative from the government and CDC has accordingly focused on this, it is perhaps not surprising that many would cling to this personal choice argument. However, based on the present results as well as accumulating evidence from the literature of reductions in infectiousness and reductions in community transmission, it may be time to refocus this narrative to recognize that the benefits of vaccination are not solely personal, but also communal as well.

## Supplementary Information

Below is the link to the electronic supplementary material.

Supplementary file1 (XLSX 239 KB)

## Data Availability

All data are drawn from publicly available sources, and provided in the supplementary materials.

## Footnotes

1 Centers for Disease Control and Prevention. United States COVID-19 Cases and Deaths by State over Time (Data Set) 2021. https://data.cdc.gov/Case-Surveillance/United-States-COVID-19-Cases-and-Deaths-by-State-o/9mfq-cb36 (Accessed Oct 29 2021).

2 Centers for Disease Control and Prevention. COVID-19 Vaccinations in the United States,Jurisdiction | Data | Centers for Disease Control and Prevention 2021. https://data.cdc.gov/Vaccinations/COVID-19-Vaccinations-in-the-United-States- Jurisdi/unsk-b7fc (Accessed Oct 30 2021).

3 Kaiser Family Foundation. COVID-19 Vaccine Breakthrough Cases: Data from the States. KFF 2021. https://www.kff.org/policy-watch/covid-19-vaccine-breakthrough-cases-data-from-the-states/ (Accessed Oct 30 2021).

4 World Population Review. US States - Ranked by Population 2021. https://worldpopulationreview.com/states (Accessed Oct 30 2021).

5 US Department of Health and Human Services. COVID-19 Diagnostic Laboratory Testing (PCR Testing) Time Series | HealthData.gov n.d. https://healthdata.gov/dataset/COVID-19-Diagnostic-Laboratory-Testing-PCR-Testing/j8mb-icvb (Accessed Oct 30 2021).

6 Bureau of Transportation Statistics. Trips by Distance | Open Data | Socrata n.d. https://data.bts.gov/Research-and- Statistics/Trips-by-Distance/w96p-f2qv (Accessed Oct 30 2021).

7 Delphi Group D. COVIDcast Export Data n.d. https://delphi.cmu.edu/covidcast/export/ (Accessed Nov 30 2021).
